# Reliability of last menstrual period recall, an early ultrasound and a Smartphone App in predicting date of delivery and classification of preterm and post-term births

**DOI:** 10.1186/s12884-021-03980-6

**Published:** 2021-07-07

**Authors:** Linda Majola, Samantha Budhram, Vani Govender, Megeshinee Naidoo, Zukiswa Godlwana, Carl Lombard, Dhayendre Moodley

**Affiliations:** 1 Department of Obstetrics and Gynaecology, School of Clinical Medicine, University of KwaZulu-Natal, 719 Umbilo Road, Congella, 4013 South Africa; 2 Centre for the AIDS Programme of Research in South Africa, 719 Umbilo Road, Congella, 4013 South Africa; 3 Biostatistics Unit, South African Medical Research Council, Tygerberg, South Africa; 4 Division of Epidemiology and Biostatistics, Department of Global Health, University of Stellenbosch, Cape Town, South Africa

**Keywords:** Estimated Date of Delivery, Last normal menstrual period recall, Ultrasound dating, ACOG smartphone app, Preterm birth misclassification

## Abstract

**Background:**

A reliable expected date of delivery (EDD) is important for pregnant women in planning for a safe delivery and critical for management of obstetric emergencies. We compared the accuracy of LMP recall, an early ultrasound (EUS) and a Smartphone App in predicting the EDD in South African pregnant women. We further evaluated the rates of preterm and post-term births based on using the different measures.

**Methods:**

This is a retrospective sub-study of pregnant women enrolled in a randomized controlled trial between October 2017-December 2019. EDD and gestational age (GA) at delivery were calculated from EUS, LMP and Smartphone App. Data were analysed using SPSS version 25. A Bland–Altman plot was constructed to determine the limits of agreement between LMP and EUS.

**Results:**

Three hundred twenty-five pregnant women who delivered at term (≥ 37 weeks by EUS) and without pregnancy complications were included in this analysis. Women had an EUS at a mean GA of 16 weeks and 3 days). The mean difference between LMP dating and EUS is 0.8 days with the limits of agreement 31.4–30.3 days (Concordance Correlation Co-efficient 0.835; 95%CI 0.802, 0.867). The mean(SD) of the marginal time distribution of the two methods differ significantly (p = 0.00187). EDDs were < 14 days of the actual date of delivery (ADD) for 287 (88.3%;95%CI 84.4–91.4), 279 (85.9%;95%CI 81.6–89.2) and 215 (66.2%;95%CI 60.9–71.1) women for EUS, Smartphone App and LMP respectively but overall agreement between EUS and LMP was only 46.5% using a five category scale for EDD-ADD with a kappa of .22. EUS 14–24 weeks and EUS < 14 weeks predicted EDDs < 14 days of ADD in 88.1% and 79.3% of women respectively. The proportion of births classified as preterm (< 37 weeks) was 9.9% (95%CI 7.1–13.6) by LMP and 0.3% (95%CI 0.1–1.7) by Smartphone App. The proportion of post-term (> 42 weeks gestation) births was 11.4% (95%CI 8.4–15.3), 1.9% (95%CI 0.9–3.9) and 3.4% (95%CI 1.9–5.9) by LMP, EUS and Smartphone respectively.

**Conclusions:**

EUS and Smartphone App were the most accurate to estimate the EDD in pregnant women. LMP-based dating resulted in misclassification of a significantly greater number of preterm and post-term deliveries compared to EUS and the Smartphone App.

## Background

A reliable expected date of delivery (EDD) is important for pregnant women in planning for a safe and uneventful delivery and critical for clinical management of obstetric emergencies [1, 2]. In an implementation study of the Intergrowth-21 gestational dating in Kenya, all respondents interviewed reported that the EDD helped them to prepare for delivery financially and logistically [3]. According to the WHO guidelines pregnant women should have at least one ultrasound (US) before 24 weeks in pregnancy to determine the gestational age (GA), EDD and identify fetal anomalies and poor fetal growth [4]. In low and middle income countries (LMIC) the availability of an ultrasound machine and ultrasound expertise are often limited to secondary and tertiary level facilities [3–6] and pregnant women attending primary health care clinics are referred to hospitals and Midwifery Outpatient Units for an US. However, long waiting times and financial constraints together with late antenatal attendance often delay an ultrasound examination to after 24 weeks of pregnancy [6].

In the case of first visit antenatal attendees, the midwife would have to depend on the accurate recall of LMP date and determine gestational age and EDD using a “pregnancy wheel”, or on symphysis-fundus height (SFH) measurements if the LMP is unknown or unreliable [7]. SFH measurements are further complicated by small for gestational age fetuses and high body mass index (BMI) [8]. Upon receipt of an US report at a subsequent antenatal visit, the midwife is required to adjust the EDD originally determined using LMP date. In the event of a major discrepancy between the LMP dating and the US report, midwives will need to ascertain which of the two measures will be more accurate. In 2016, the American College of Obstetricians and Gynecologists (ACOG) released a new EDD Calculator smartphone application (Smartphone App) that reconciles discrepancies in due dates between the first ultrasound and the date of the LMP [1].

Previous studies comparing second trimester ultrasound with LMP dating found ultrasound, on average, resulted in lower GA estimates [9, 10]. A recent South African study compared dating by LMP, SFH and EUS and concluded that pregnancy dating by ultrasound, including those in more advanced pregnancy than currently permitted, is recommended since all non-ultrasound based estimations of GA were considerably less accurate [11].

In clinical trials and pregnancy surveillance registries investigating safety and efficacy of new treatment regimens in preventing infection and adverse pregnancy outcomes, preterm births as defined by GA < 37 weeks and severe preterm (GA < 34 weeks) births are primary clinical outcomes [12, 13]. Many multi-country pregnancy clinical trials and pregnancy surveillance protocols in LMICs depend on GA dating using the LMP and only confirmed by ultrasound, when available [14]. As a result, inconsistencies in GA assessment within and between studies could lead to variable and incomparable outcomes [15–17]. There are several smartphone apps for parents-to-be and these apps are intended to be informative for better pregnancy outcomes. There are no validity studies of pregnancy dating apps for service providers.

In this retrospective data analysis, we evaluated the accuracy of LMP recall, an EUS and the ACOG smartphone app in predicting the EDD in a South African cohort of pregnant women not living with Human Immunodeficiency Virus (HIV). We further compared the preterm and post-term birth rate as determined by each method.

## Methods

This is a secondary analysis of data collected in a randomized controlled trial (RCT) that was designed to determine the safety of Tenofovir disoproxil fumarate/Emtricitabine (TDF/FTC) in pregnancy when used as pre-exposure prophylaxis in healthy pregnant women not living with HIV (ClinicalTrials.gov NCT03227731). The RCT is being conducted at a Research Clinic in Durban, South Africa (SA) and enrolled a total of 540 pregnant women between October 2017 and December 2019. Women were 18 years or older, confirmed pregnant, confirmed testing negative for HIV infection, booked for antenatal care < 24 weeks of gestation, without life-threatening co-morbidities and at minimal risk for obstetric complications. HIV seronegative pregnant women booking for antenatal care at local primary health clinics in Umlazi, a peri-urban township in Durban, were referred to the Umlazi Research Clinic based at the Prince Mshyeni Memorial Hospital (PMMH), Durban if they were interested in participating in the parent RCT. Antenatal screening including gestational age determination was done at the research clinic. Gestational age was determined by recall of last menstrual period date and an ultrasound. Women who were eligible and consented to participation in the RCT continued with the antenatal care provided at the research clinic. Women delivered at PMMH and continued with postpartum care at the Research Clinic.

For this sub-study, the following exclusion criteria were applied:

- the recollection of the date of the LMP was unknown or deemed unreliable
- planned cesarean delivery
- induction of labour prior to 37 weeks
- preterm delivery (< 37 weeks based on EUS dating) occurred
- there was a diagnosis of polyhydramnios, abruptio placentae
- multiple pregnancy, intra-uterine growth restriction and/or fetal anomaly
- insufficient clinical notes

At screening for the parent study, all pregnant women underwent an obstetric ultrasound examination, using the Portable PC-Based TECHNO 8000 Ultrasonic Diagnostic Instrument, at the research clinic. Upon confirmation of a viable intra-uterine pregnancy, standard fetal biometry (a crown-rump length (CRL) in the first trimester and a biparietal diameter (BPD Outer-Inner), abdominal circumference (AC) and femur length (FL) in the second trimester were measured. With these measurements, the EDD and the average GA was also expressed in the report [18]. The ultrasound was performed by a single sonographer. In addition to an obstetric and general examination, the pregnant woman also provided the date of her last LMP, if known. For this sub-study analysis, an EDD was calculated from the EUS, LMP and the Smartphone App. The ACOG smartphone app can be downloaded from the ACOG website (https://www.acog.org/membership/member-benefits/acog-app) or from Google play (https://play.google.com/store/apps/details?id=vspringboard.acog.activity). The App is free of charge and does not need internet access. Among other features on the App, the “Estimated Due Date Calculator” based on guidance from ACOG and others, uses data from last menstrual period and first accurate ultrasound to determine estimated due date and target date for gestational age. The App has the ability to reconcile the discrepancy in due dates between the ultrasound-determined dates and the date of the last menstrual period as and when redating is recommended [1]. Further guidance to redating is provided in the ACOG Committee Opinion and is based on the timing of the ultrasound and the discrepancy between LMP and US dating [1].

Delivery data such as actual date of delivery (ADD), mode of delivery and birth weight were extracted from the hospital records. GA at delivery was calculated using the EUS, LMP and the ACOG Smartphone App independent of each other.

### Definitions

Reliable LMP: By convention, pregnancies are dated in weeks starting from the first day of a woman's last LMP. The questions that were routinely asked at the research clinic when determining reliability of the LMP were:

- have you had regular menstrual periods over the last 6 months
- were you on any hormonal therapy including contraceptive in the last 6 months
- were you breast feeding in the last 6 months
- were you pregnant in the last 6 months
- was the conception spontaneous

EUS: Early obstetric ultrasound is an ultrasound examination before 24 weeks gestation.

EDD based on LMP: The estimated/expected date of delivery, also known known as estimated due date or simply known as due date, is a term describing the estimated date of confinement usually corresponding to 40 weeks (279 days) from the 1^st^ day of the last normal menstrual period.

EDD based on EUS: The estimated/expected date of delivery corresponding to 40 weeks from the gestational age determined by EUS.

ADD: Actual date of delivery.

GA at Delivery in weeks and days: Is calculated using the following formula: 280 days – (EDD-ADD).

Preterm Birth: gestation < 37 complete weeks.

Post-term Birth: gestation > 42 complete weeks.

### Ethics approval and consent to participate

The Institutional Review Board of University of KwaZulu-Natal approved the study (Ref BE 00000817/2019, a sub-study of BFC 243/16). For this retrospective data analysis of de-identified data, a written informed consent was waived by the Institutional Review Board of University of KwaZulu-Natal.

Data were analysed using SPSS version 25. Descriptive statistics such as frequencies and percentages were used to summarise categorical variables. Central tendency and dispersion of data were measured using means and standard deviations for normally distributed variables and medians and interquartile ranges for skewed variables. A p-value less than 0.05 was considered statistically significant. We evaluated the concordance of the LMP, EUS and the ACOG app in predicting the expected date of delivery and calculated the concordance correlation coefficient (ccc) with 95% confidence intervals as well as the Bland–Altman limits of agreement [19]. We also categorized the EDD-ADD difference into five intervals (≤ -15, -14 to -8, -7 to 7, 8 to 14, ≥ 15 days) and used the kappa statistic to evaluate the agreement between the LMP and EUS as a robust measure since the distribution of the EDD-ADD difference of the two measures were different.

## Results

Of the 540 women enrolled in the parent study, 325 women met the eligibility criteria for the sub-study analysis. The 325 women had an ultrasound performed at a mean GA of 16 weeks and 2 days, and all had an EUS (< 24 weeks). The mean (SD) age of the women was 23.7 (4.5) years with the majority (79.1%) having an education level of matriculation or higher (Table 1). The mean (SD) BMI was 27.5 kg/m^2^ (5.8) and 31% of the cohort were obese (BMI ≥ 30 kg/m^2^). The mean (SD) birthweight of the neonate was 3200 g (400). The median estimated GA at birth was 277 days (week 39 + 4), 276 days (week 39 + 3) and 277 days (week 39 + 4) by EUS, LMP and Smartphone App respectively.

**Table 1:** Study population characteristics by 1^st^ and 2^nd^ trimester antenatal booking (*N* = 325)

	**1**^**st**^** Trimester (< 14 weeks) *n* = 82**	**2**^**nd**^** Trimester (14 to 28 weeks) *n* = 243**	***P* Value**
**Gestational Age (weeks)** Mean (SD)	10.33 (2.2)	18.4 (2.9)	-
**Age (years)** Mean (SD) **Category n (%)**	23.7 (4.2)	23.7 (4.7)	0.988
< 20	13 (15.9)	46 (18.9)	0.621
20 – 30	61 (74.4)	167 (68.7)	
≥ 30	8 (9.8)	30 (12.4)	
**Education n (%)**			
Grade 8–11	10 (14.7)	58 (85.3)	
Matriculation and Higher	72 (28.0)	185 (71.9)	0.016*
**Parity** Mean (SD) **Category n (%)**	1.7 (0.8)	1.8 (1.2)	0.510
1	42 (51.2)	120 (49.4)	
2–3	38 (46.3)	110 (45.3)	0.554
> 3	2 (2.4)	13 (5.4)	
**Body Mass Index (kg/m**^**2**^**)** Mean (SD) **Category n (%)**	27.7 (6.1)	27.4 (5.7)	0.745
** < 30**	47 (63.5)	151 (70.9)	
** > 30**	27 (36.5)	62 (29.1)	0.150
**Mode of Delivery n (%)**			
Spontaneous Vaginal	59 (23.2)	195 (76.8)	0.079
Caesarean section after labour	23 (32.4)	48 (67.6)	
**Birthweight (g)**			
Mean (SD)	3142 (372)	3165 (413)	0.665
**Estimated Gestational Age (days) at delivery by ultrasound**			
Mean (SD)	276 (9)	277 (8)	0.092
**Estimated Gestational Age (days) at delivery by LMP**			
Mean (SD)	277 (16)	276 (19)	0.626

**Fig. 1:**
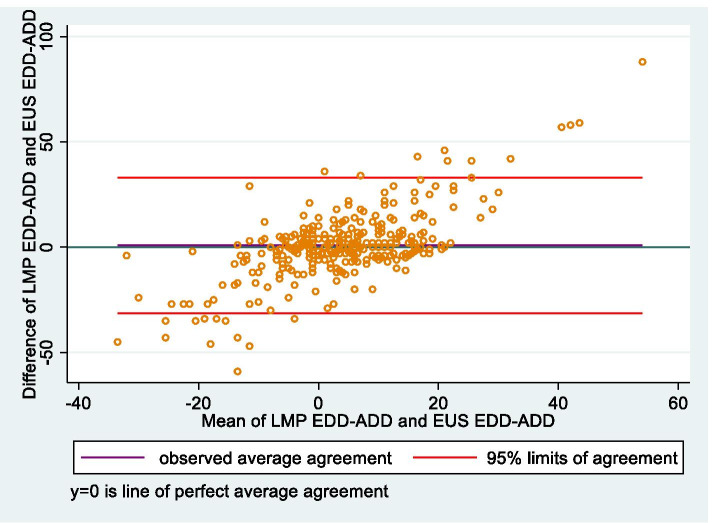
Limits of agreement between LMP and EUS (Bland JM, Altman DG (1986)

The mean number of days from initial antenatal assessment to expected delivery was 148.2 ± 30.1 (range 45 – 235) and 147.4 ± 27.1 (range 79 – 237) days for LMP and EUS dating respectively. Figure 1 shows a Bland Altman plot for the two methods LMP and EUS (Concordance Correlation Co-efficient 0.835; 95% CI 0.802, 0.867). The mean difference between LMP dating and EUS is 0.8 days with the limits of agreement 31.4 to 30.3 days. The mean and SD of the marginal time distribution of the two methods differ significantly (*p* = 0.00187).

The median estimated GA at birth was 277 days (week 39 + 4) and 276 days (week 39 + 3) gestation for the EUS and LMP dating respectively. Compared to LMP, EUS derived GA at delivery was slightly longer (mean of 277 days vs. 276 days), less variable (standard deviation of 8 days vs. 18 days) and had a narrower range (247 – 308 days vs. 220 – 374 days). The proportion of births classified as preterm (< 37 weeks gestation) was 9.9% (95%CI 7.1 – 13.6) by LMP and 0.3% (95%CI 0.1–1.7) by ACOG Smartphone App. The proportion of post-term (> 42 weeks gestation) births was 11.4% (95%CI 8.4–15.3), 1.9% (95%CI 0.9–3.9) and 3.4% (95%CI 1.9–5.9) by LMP, EUS and Smartphone respectively. Labour was induced in 24 (7.4%) women, this including one post term birth and 23 term births as classified by EUS.

**Fig. 2:**
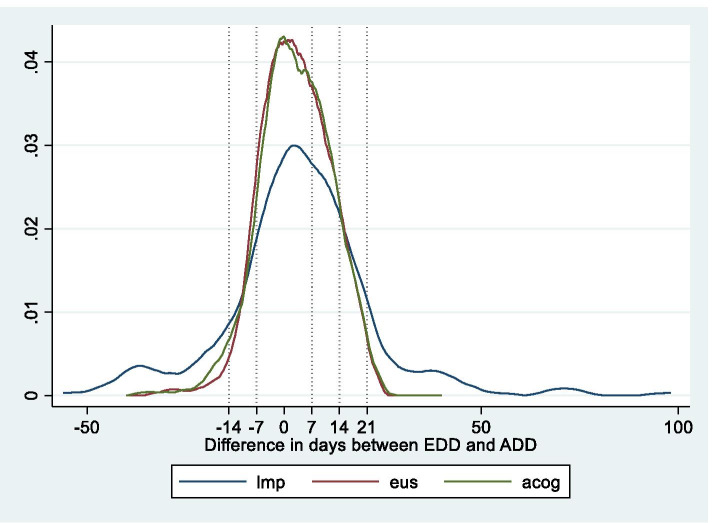
Kernel density estimates of deviation of the actual delivery date from the estimated delivery date of delivery calculated from an ultrasound (EUS), last menstrual period (LMP) and ACOG App

EDDs were within 14 days of the ADD for 287 (88.3%; 95%CI 84.4–91.4), 279 (85.9%; 95%CI 81.6–89.2) and 215 (66.2%; 95%CI 60.9–71.1) women for EUS, ACOG Smartphone App and LMP respectively (Table 2). An EUS performed 15 – 24 weeks gestation predicted EDDs within 14 days of ADD in 89.7% (95%CI 85.2–93.2) of women versus 84.2% (95%CI 74.4–91.3) (OR 0.61; 95%CI 0.29–1.25; *p* = 0.125) with an EUS performed before14 weeks (Table 2). A comparison of the difference in days between ADD and EDD for LMP, EUS and ACOG Smartphone app using the Bland Altman analysis suggest a poor concordance correlation between LMP and EUS (ccc r = 0.35; 95%CI 0.28–0.41). There was also poor agreement in the difference of days (ADD-EDD) between LMP and EUS when comparing the categorical distribution using 7-day intervals (overall agreement = 46.5% with kappa = 0.22 with SE = 0.03) (Table 3 and Fig. 2).

**Table 2:** Predictive accuracy of the Estimated Date of Delivery (EDD) for the Actual Date of Delivery (ADD) for 7 and 14 day windows

	**Using LMP alone** ***n* = 325**	**Using EUS alone (< 24 weeks)** ***n* = 325**	**Using EUS alone** **(< 14 weeks)** ***n* = 82**	**Using EUS alone** **(14 – 24 weeks)** ***n* = 243**	**Using the Smartphone App *n* = 325**
**EDD within 7 days of ADD** **N (%) (95%CI)**	134 (41.2) (36.0–46.7)	193 (59.4) (53.9–64.6)	41 (50) (38.8–61.3)	152 (62.6) (56.1–68.7)	185 (56.9)
**EDD within 14 days of ADD** **N (%) (95%CI)**	207 (63.7) (60.9–71.1)	287 (88.3) (84.4–91.4)	69 (84.2) (74.4–91.3)	218 (89.7) (85.2–93.2)	279 (85.9) (81.6–89.2)

**Table 3:** Cross-tabulation of categorized EDD-ADD differences for LMP and EUS (cell frequencies and percentages)

**LMP**	**EUS**					
	≤ -15 days	-14 to -8 days	-7 to 7 days	8 to 14 days	≥ 15 days	TOTAL
≤ -15 days	3 **0.92**	10 3.08	23 7.08	3 0.92	1 0.31	40 12.31
-14 to -8 days	1 0.31	6 **1.85**	15 4.62	1 0.31	2 0.62	25 7.69
-7 to 7 days	2 0.62	5 1.54	104 **32.00**	21 6.46	2 0.62	134 41.23
8 to 14 days	0 0.00	1 0.31	23 7.08	14 **4.31**	10 3.08	48 14.77
≥ 15 days	1 0.31	1 0.31	28 8.62	24 7.38	24 **7.38**	78 24.00
TOTAL	7 2.15	23 7.08	193 59.38	63 19.38	39 12.00	325 100.00

Overall observed agreement 46.5%, expected overall chance agreement 31.0%

## Discussion

In this sub-study of healthy pregnant women not living with HIV, we found that EDDs derived from an EUS performed < 24 weeks gestation and using the ACOG Smartphone App were both within 2 weeks of actual delivery in more than 85% of the study population as opposed to LMP derived EDDs in 66% of the population. The mean EDD is very similar between the methods but at an individual participant level the agreement is poor with limits of agreement > 30 days which indicates large differences given the short time frame of pregnancy. In addition, we report the similar marginal predictive accuracy of EDDs within 2 weeks of ADD (-14 to 14 days) for LMP and EUS (84.2% vs 89.7%) but when comparing categorized EDD-ADD difference head to head the agreement is also poor (kappa = 0.22). Estimated GA at birth derived from the LMP resulted in 10% of births to be misclassified as preterm (< 37 weeks) and 11% post-term (> 42 weeks). There was one preterm birth with EDD determined on the ACOG Smartphone App.

Several studies in the past decade have assessed the accuracy of LMP dating in comparison to an ultrasound [11, 12, 20–25] and none that evaluated the use of a Smartphone app. In the majority of the studies, LMP is less reliable because of poor recall, irregular menstrual cycles and prior use of hormonal contraception [7, 26–28]. Our findings of the mismatch between EDD and ADD using LMP dating alone in this healthy cohort of pregnant women are not unique but merely strengthen the need for an ultrasound assessment as an essential obstetric service in LMICs and as per WHO recommendations [4]. Consistent with our findings, the use of ultrasound dating correctly predicted date of delivery within 14 days of actual delivery in 85% and 91% of the study cohort in two other studies respectively [11, 29]. In these studies, use of a paper pregnancy wheel to calculate gestational age and EDD could have also contributed to the weaker ability to correctly predict the EDD based on LMP dating [30, 31]. These studies have demonstrated inconsistencies in determining EDDs using paper pregnancy wheels of various types. Many of these pregnancy wheels deviated from the standard pregnancy duration of 280 days [30]. The ACOG smartphone app offers the option of using LMP dating alone in the absence of an ultrasound, or an ultrasound alone or a combination of both methods. Using smartphone technology that is widely available in most LMIC countries, and ultrasound dating when available would provide women and clinicians more accurate estimates of expected date of delivery and could reduce maternal and perinatal morbidity associated with non-facility based deliveries.

The implications of an inaccurate EDD are two-fold. As an intervention to minimize the number of non-facility based deliveries and associated adverse pregnancy outcomes, women in rural resource limited settings are advised to pre-arrange suitable transport to their nearest maternity hospital in advance of their due date for delivery. In a study of more than 4000 women in Zanzibar, 28% of EDDs were overestimated ie. beyond the ADD and as a result these women were less likely to deliver at a health facility [28]. Not delivering at a health facility or delay in presenting to the health facility when in labour has a major impact on perinatal and maternal morbidity and mortality. The other clinical implication of inaccurate EDDs derived from LMP lies in the misclassification of preterm and post-term births. In our study, using LMP in the absence of an EUS would have misclassified 10% of the term births as preterm (< 37 weeks) and 11% as post-term (> 41 weeks). Other studies have reported similar misclassifications [17, 25, 32]. More importantly, such misclassifications have also been reported in large scale surveillance reports. A prospective study carried out in the Western Cape, SA to elucidate reasons for inconsistent associations between antiretroviral use and preterm birth had similar findings to our study with there being a significantly greater number of pre- and post-term deliveries in women who were not dated by ultrasound and that ultrasound-based dating was more accurate in predicting spontaneous labour than LMP or SFH based dating, both individually or in combination [33]. Tunon et al. compared ultrasound to a reliable LMP in predicting the ADD in 15 000 women and showed that there was a significantly narrower distribution of births according to the ultrasound estimates and the proportion post-term births were significantly greater by LMP versus ultrasound dating methods [34]. A more recent study conducted in Johannesburg, SA, concluded that in the absence of ultrasound, LMP is a reliable alternative for GA dating during early pregnancy [11]. However, it is not sensitive in identifying late- and post-term pregnancies and should not be relied upon to make clinical decisions regarding elective cesarean section or induction of labor for supposed prolonged pregnancies.

Our study findings highlight a potential preventable factor in reducing perinatal morbidity and mortality. In the absence of dating by EUS, pregnancies may complicate with the potential to contribute to the burden of prematurity, unnecessary inductions of labour in already resource-constrained settings and undiagnosed post-term pregnancies with its attendant risks of perinatal morbidity and mortality. Additionally, the largest category of perinatal deaths in SA is unexplained stillbirth, of which up to one-quarter have intrauterine growth restriction [14], another risk that may be modified by improved pregnancy dating.

The strength of our study lies in the head-to-head comparison of LMP and EUS dating in a healthy pregnant population. This is the first study that evaluates a Smartphone app in a research setting. While we are aware that an US report may or may not be available some time after the 1^st^ antenatal visit in resource limited settings, the Smartphone app should be further evaluated in the primary health care setting where there is an expected discrepancy in EDD between LMP dating at the 1^st^ visit and an US report at later visits. For comparative purposes, our smaller sample size is limiting. Our other limitation is the absence of SFH measurements however the high proportion of obesity in our local population could likely not have been reliably measured in almost 30% of the population.

## Conclusions

EUS alone and the Smartphone App method of pregnancy dating are the most accurate methods to estimate the EDD in a cohort of healthy South African pregnant women. The use of LMP-based dating method results in the misclassification of a significantly greater number of preterm and post-term deliveries compared to EUS and Smartphone App dating methods.

## Data Availability

The datasets used and/or analysed during the current study available from the corresponding author on reasonable request.

## Abbreviations

GA: Gestational Age
EDD: Expected Date of Delivery
ADD: Actual Date of Delivery
US: Ultrasound
EUS: Early Ultrasound
LMP: Last Menstrual Period
LMIC: Low and Middle Income Country
SFH: Symphysis Fundal Height
BMI: Body Mass Index
RCT: Randomised Control Trial
TDF/FTC: Tenofovir diphosphate fumarate/Emtricitabine
